# Prophylactic Antibiotics in Vertebroplasty and Kyphoplasty: A Nationwide Analysis of Infection Rates and Antibiotic Use in South Korea

**DOI:** 10.3390/antibiotics14090901

**Published:** 2025-09-05

**Authors:** Youngjin Kim, Young-Hoon Kim, Sukil Kim, Jun-Seok Lee, Sang-Il Kim, Joonghyun Ahn, So-Young Han, Hyung-Youl Park

**Affiliations:** 1 Department of Preventive Medicine and Public Health, College of Medicine, The Catholic University of Korea, Seoul 06591, Republic of Korea; yj.kim026@catholic.ac.kr (Y.K.);; 2 Department of Orthopedic Surgery, Seoul St. Mary’s Hospital, College of Medicine, The Catholic University of Korea, Seoul 06591, Republic of Korea; boscoa@catholic.ac.kr (Y.-H.K.);; 3 Department of Orthopedic Surgery, Eunpyeong St. Mary’s Hospital, College of Medicine, The Catholic University of Korea, Seoul 03312, Republic of Korea; 4 Department of Orthopedic Surgery, Bucheon St. Mary’s Hospital, College of Medicine, The Catholic University of Korea, Bucheon 14647, Republic of Korea; 5 Healthcare Review and Assessment Committee, Health Insurance Review & Assessment Service, Wonju 26465, Republic of Korea

**Keywords:** antibiotic prophylaxis, spine surgery, vertebroplasty, kyphoplasty, surgical wound infection

## Abstract

**Background/Objectives**: Vertebroplasty (VP) and kyphoplasty (KP) are widely performed minimally invasive procedures for osteoporotic vertebral compression fractures and vertebral metastases. Although generally safe, postoperative surgical site infections (SSIs) can lead to severe complications. The true incidence of SSIs and optimal prophylactic antibiotic strategies remains unclear. This study evaluated SSI incidence and the impact of antibiotic timing and type using a nationwide quality assessment (QA) database in South Korea. **Methods**: We analyzed data from the 7th to 9th QA waves of the Health Insurance Review and Assessment (HIRA) Service, including 23,868 patients who underwent VP or KP. SSI incidence was compared across antibiotic timing groups (preoperative-only, postoperative-only, and combined) and antibiotic types. Multivariate logistic regression identified independent risk factors for SSIs. **Results**: SSI occurred in 47 patients (0.20% of 23,868 procedures). No infections were observed in the preoperative-only group, compared with 0.36% in the postoperative-only group and 0.19% in the pre- and postoperative group. The lowest incidence (0.16%) was seen with first- or second-generation cephalosporins. Multivariate analysis found no significant difference between the preoperative-only and the combined regimens, nor between first-/second-generation cephalosporins and broad-spectrum antibiotics. However, surgery at a tertiary hospital (aOR: 3.566) and malnutrition (aOR: 2.915) were independently associated with increased SSI risk. **Conclusions**: This nationwide study, the largest to date on VP and KP, demonstrated that SSIs are rare (0.2%). A single preoperative dose of first- or second-generation cephalosporins was as effective as combined or broader-spectrum regimens. Targeted preventive measures may be warranted for high-risk groups such as patients with malnutrition or those treated in tertiary hospitals.

## 1. Introduction

Vertebroplasty (VP) and kyphoplasty (KP) are widely performed minimally invasive procedures for osteoporotic vertebral compression fractures and vertebral metastases [1,2]. These procedures provide rapid pain relief, restore spinal stability, and improve functional outcomes, thereby enhancing patients’ quality of life [3,4,5,6]. Although generally considered safe, infectious complications such as vertebral osteomyelitis, discitis, and epidural abscesses can occur, often resulting in severe morbidity, prolonged hospitalization, and the need for revision surgery [7,8].

The precise incidence of surgical site infections (SSIs) after VP and KP remains uncertain, as most previous studies were limited to small-scale or single-center cohorts with heterogeneous methodologies [9,10,11]. Consequently, there is no clear consensus on whether prophylactic antibiotics are necessary, and if so, what the timing and regimen should be. Current practice varies widely across institutions, while guideline recommendations for spinal procedures have not been specifically validated in the context of VP and KP [12].

In South Korea, the Health Insurance Review and Assessment (HIRA) Service, a government-affiliated agency operating under the National Health Insurance system, conducts nationwide quality assessment (QA) programs to monitor prophylactic antibiotic use in surgical procedures [13]. Unlike insurance claims databases that rely solely on diagnostic codes, the QA dataset provides standardized information on antibiotic administration and infection outcomes [14,15]. This allows for a more accurate evaluation of SSI incidence and risk factors, addressing key limitations of earlier studies.

Using this nationwide QA database, we aimed to determine the incidence of SSIs following VP and KP and to evaluate the effectiveness of prophylactic antibiotic strategies in a large, real-world cohort. To our knowledge, this is the first nationwide analysis focused specifically on these procedures. By validating prior smaller-scale observations at the national level, this study provides robust real-world evidence to refine prophylactic antibiotic strategies and guide infections prevention practices in minimally invasive spinal surgery.

## 2. Results

### 2.1. Incidence of Postoperative Infections

Among the 23,868 patients who underwent VP or KP, 47 cases of SSI were identified (overall incidence, 0.20%). The incidence was 0.21% in the VP group (43 cases) and 0.13% in the KP group (4 cases), with no significant difference between procedures (*p* = 0.3872). Non-surgical site infections occurred in 364 cases (1.53%), resulting in a total postoperative infection rate of 1.71% (407 cases) (Table 1).

### 2.2. Baseline Characteristics by Timing of Prophylactic Antibiotic Use

Baseline characteristics are summarized in Table 2, with full details provided in Supplementary Table S1. Patients in the preoperative-only group (*N* = 1131) were significantly older than those in the postoperative-only group (77.62 ± 8.48 vs. 76.68 ± 8.37 years, *p* < 0.001). Diabetes mellitus (DM) (34.31% vs. 30.17%) and hypertension (52.08% vs. 41.91%) were also more prevalent in the preoperative-only group (both *p* < 0.05).

There were no significant differences among groups in malnutrition (3.89%, 2.81%, and 3.00%, respectively, *p* = 0.188), uncontrolled diabetes (0.80%, 0.51%, and 0.53%; *p* = 0.497), or skin/soft tissue infection (4.60%, 4.39%, and 5.01%; *p* = 0.349).

Regarding antibiotic selection, first- or second-generation cephalosporins were used most frequently in the preoperative-only group (94.78%), compared with 87.35% in the postoperative-only group and 89.56% in the pre- and postoperative group (*p* < 0.001).

### 2.3. Postoperative Infection Rates by Timing of Prophylactic Antibiotic Use

**Figure 1:**
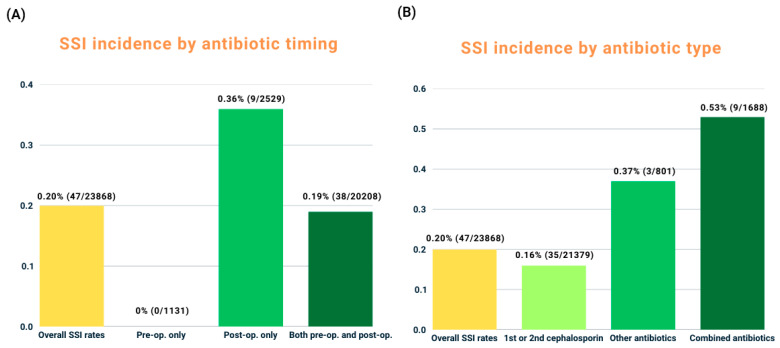
Distribution of patients and corresponding SSI incidence according to (**A**) timing of prophylactic antibiotic administration and (**B**) type of prophylactic antibiotics.

No SSIs occurred in the preoperative-only group, whereas the postoperative-only group had the highest incidence at 0.36% (9 cases), followed by 0.19% (38 cases) in the pre- and postoperative group. The difference among the three groups was not statistically significant (*p* = 0.069) (Table 3, Figure 1A).

### 2.4. Postoperative Infection Rates by Type of Prophylactic Antibiotics Used

First- and second-generation cephalosporins were the most common prophylactic agents in all groups, particularly in the preoperative-only group. A detailed distribution of antibiotic classes by timing of administration is provided in Supplementary Table S2. The lowest SSI incidence was observed in patients who received only first- or second-generation cephalosporins (0.16%, 35 cases), followed by those who received only other antibiotics (0.37%, 3 cases). The highest incidence occurred in patients who received both cephalosporins and other antibiotics (0.53%, 9 cases). The difference was statistically significant (*p* = 0.004) (Table 4, Figure 1B).

### 2.5. Multivariate Analysis of Surgical Site Infections

Results of multivariate logistic regression are presented in Table 5. Compared with pre- and postoperative prophylaxis, preoperative-only administration was associated with lower odds of SSI (adjusted odds ratio (OR): 0.270; 95% confidence interval (CI): 0.021–3.554), though not statistically significant. Similarly, use of first- or second-generation cephalosporins showed lower odds compared with other antibiotics (aOR: 0.921; 95% CI: 0.252–3.364), with statistical significance.

Independent predictors of SSI included surgery at a tertiary hospital (aOR: 3.566; 95% CI: 1.017–12.502) and malnutrition (aOR: 2.915; 95% CI: 1.129–7.522).

## 3. Discussion

The HIRA service in Korea oversees healthcare quality through a nationwide medical claims system, enabling comprehensive quality assessments and incentivizing hospitals to comply with established QA standards [16,17]. Since 2007, HIRA has conducted nine QA waves evaluating prophylactic antibiotic use across various surgical procedures [13,14]. Our study utilized this dataset, which covers all Korean hospitals and provides standardized records of prophylactic antibiotic administration and postoperative infections within three months of surgery. This comprehensive scope allowed for a robust evaluation of SSI incidence after VP and KP, addressing the limitations of previous smaller-scale or single-center studies.

Although VP and KP are minimally invasive procedures, the risk of infection remains, as with any percutaneous surgical intervention [18]. Postoperative infections such as vertebral osteomyelitis or discitis may result from preexisting spondylitis or from procedure-related contamination [19,20]. In Korea, mandatory conservative management periods (two weeks for VP, three weeks for KP) before surgery help exclude patients with preexisting infections [21]. Moreover, by strictly excluding cases with documented preoperative infections, our study specifically isolated procedure-related SSIs. This methodological rigor strengthens the reliability and clinical applicability of our findings.

In this nationwide cohort, SSIs occurred in 47 of 23,868 VP or KP procedures (0.20%), confirming that infection is a rare complication. This rate is lower than previously reported, 0.46% by Abdelrahman et al. [9] and 0.36% by Park et al. [10], and reinforces the overall safety of VP and KP [22]. Importantly, our analysis represents the largest cohort study to date, providing robust real-world evidence to guide infection prevention practices in these procedures.

The relatively low incidence observed in our study may partly reflect Korea’s practice of mandatory conservative management, which likely excludes patients with occult infections before surgery. While this strengthens the internal validity of our findings, it may limit generalizability to countries without such protocols, such as the United States or many European nations [23]. Therefore, differences in baseline patient selection and procedure timing should be considered when applying our results to international practice.

Currently, no specific guidelines exist for prophylactic antibiotic regimens in VP and KP [12,24]. The North American Spine Society (NASS) guidelines broadly recommend a single preoperative dose with additional intraoperative dosing as needed for spine surgery, but they do not specifically address vertebral augmentation [12]. Our findings directly support preoperative-only prophylaxis, aligning with these broader recommendations.

Remarkably, despite the preoperative-only group having less favorable baseline characteristics, including older age, higher prevalence of diabetes and hypertension, and more frequent tertiary hospital admissions, no SSIs were observed in this group. This suggests that the timing of antibiotic administration, specifically preoperative coverage, may play a critical role in preventing infection, even in higher-risk populations. Multivariate analysis confirmed that preoperative-only prophylaxis was associated with lower odds of SSIs compared with pre- and postoperative prophylaxis, though the difference was not statistically significant. Taken together, these findings suggest that a single, well-timed preoperative dose may be as effective as perioperative combinations in preventing SSIs.

In contrast, a prior study by Chen et al. [11], in which prophylactic antibiotics were not routinely administered, reported a significantly higher SSI incidence of 1.26% (9 cases among 716 patients), compared with 0.20% in our cohort. These findings support the protective role of preoperative antibiotics in reducing infection risk [12]. Moreover, our analysis indicated that administering antibiotics only after surgery was associated with markedly higher odds of SSIs compared to combined prophylaxis (aOR: 24.806). This increased risk is likely attributable to the absence of bactericidal coverage at the time of incision. When antibiotics are delayed until after surgery, intraoperative contamination may occur before adequate antibiotic levels are achieved, thereby negating the preventive purpose of prophylactic administration [25]. Taken together, these findings suggest that either omitting antibiotics entirely or delaying administration until after surgery provides no meaningful protection against infection [26].

Our findings also highlight the impact of antibiotic selection on infection risk. The lowest SSI incidence (0.16%) was observed with first- and second-generation cephalosporins, whereas broader-spectrum antibiotics alone showed an SSI rate of 0.30%, and combination antibiotic regimens (cephalosporins plus other agents) showed the highest rate at 0.53%. Multivariate analysis showed no significant difference in SSI risk between patients who received only first- or second-generation cephalosporins and those who received broader-spectrum antibiotics (aOR: 0.921; CI: 0.252–3.364), indicating that broader-spectrum agents do not provide additional benefits in preventing infections. These findings support the use of first- and second-generation cephalosporins as the most appropriate prophylactic choice in VP and KP, consistent with current guideline recommendations and reinforcing the effectiveness of narrow-spectrum agents [27,28].

Interestingly, patients who received combination antibiotic regimens demonstrated the highest SSI rate (0.53%) despite broader coverage, as well as a markedly higher incidence of non-surgical site infections (13.03%). This pattern likely reflects confounding by indication, in which patients perceived as being at higher risk were more likely to receive multiple or broader-spectrum antibiotics, rather than the regimens themselves increasing infection risk. While this interpretation should be made with caution, it suggests the complexity of antibiotic selection in real-world clinical practice.

In addition to systemic prophylactic antibiotics, local antibiotic delivery methods may provide additional benefits for high-risk patients. However, in Korea, antibiotic-loaded bone cement has not been applied to VP or KP procedures and was therefore not included in our evaluation of prophylactic antibiotic strategies. Antibiotic-loaded formulations, such as gentamicin- or vancomycin-loaded cement, can achieve sustained high local antibiotic concentrations with reduced systemic toxicity, and their efficacy in infection prevention has been well established in arthroplasty and extremity trauma [29]. Although evidence in spine surgery remains limited, emerging reports suggest that antibiotic-loaded bone cement in KP or VP may help reduce infection risk. Nevertheless, concerns regarding cytotoxicity, impaired bone healing, cement weakening, and antibiotic resistance highlight the need for further high-quality studies before routine adoption in spinal procedures [30,31].

Our multivariate analysis further identified surgery performed at a tertiary hospital (aOR: 3.566; 95% CI: 1.017 to 12.502) and malnutrition (aOR: 2.915; 95% CI: 1.129 to 7.522) as independent risk factors for SSI. These results suggest that patients treated at tertiary centers, who often present with greater clinical complexity, may require closer perioperative management [32]. Notably, malnutrition showed a stronger association with infection risk than more common conditions such as diabetes or hypertension [33]. These results emphasize the importance of nutritional assessment and targeted preventive strategies in vulnerable patient populations.

This study has several limitations. First, its retrospective observational design precludes establishing definite causal relationships between prophylactic strategies and infection outcomes. Such a design is also subject to unmeasured confounding, indication bias in antibiotic selection, and institutional variations in practice, while local antibiograms data were not available. Second, because SSIs were rare (0.20%) and absent in the preoperative-only group, we used Firth’s penalized logistic regression to address small-sample bias. Nevertheless, some subgroup analyses yielded wide confidence intervals and limited statistical power, requiring cautious interpretation. Third, QAs recorded only infections diagnosed during the index hospitalization or within a 3-month follow-up, which may have underestimated late-onset SSIs or antibiotic-related adverse events such as resistance [10,20]. Fourth, reliance on the nationwide QA database restricted our ability to evaluate detailed clinical variables, including fracture etiology, smoking or alcohol history, body mass index, pulmonary or cardiac comorbidities, and use of immunosuppressive agents. Malnutrition was defined by diagnostic coding and primarily reflected in patients being underweight or having protein-calorie deficiency, while obesity was not captured. Finally, although the dataset underwent standardized QA processes, the possibility of underreporting or incomplete documentation cannot be entirely excluded. Nonetheless, the structured QA framework likely reduced these errors and improved data reliability.

Future research should include prospective multicenter studies with larger sample sizes, richer clinical detail, and longer follow-up to validate our findings, better define patient-level risk factors, and optimize prophylactic antibiotic strategies for minimally invasive vertebral procedures.

Despite these limitations, this study represents the largest and most comprehensive assessment of SSI rates after VP and KP to date, demonstrating that the incidence remains consistently low. Our findings provide strong evidence supporting preoperative prophylaxis, particularly with first- or second-generation cephalosporins, as an effective and pragmatic strategy. In addition, this work validates prior smaller-scale observations within a nationwide cohort, thereby reinforcing current guideline recommendations with large-scale real-world evidence.

## 4. Materials and Methods

### 4.1. Data Sources

The HIRA has conducted nine nationwide QA waves on prophylactic antibiotic use in surgical procedures from 2007 to 2020. The QA database includes standardized data on antibiotic administration and postoperative infection outcomes, providing more accurate assessment than datasets based solely on the International Classification of Diseases, 10th Revision (ICD-10) codes [13,14]. This reduces the risk of misclassification that often affects large administrative databases [34].

To enhance data completeness, the QA database was linked to the HIRA health insurance claims database using anonymous join keys, enabling the assessment of comorbidities and medical history not captured within the QA dataset alone [35]. This study was approved by HIRA (approval no. M20230221003) and by the Institutional Review Board of Eunpyeong St. Mary’s Hospital, Catholic University of Korea (approval no. PC23ZISI0031).

### 4.2. Study Population

All patients who underwent VP or KP during the QA waves were eligible. We analyzed the 7th QA (September–November 2015), 8th QA (October–December 2017), and 9th QA (October–December 2020). Although spinal procedures were first included in the 6th QA, only 410 cases were recorded, which was insufficient for reliable analysis. Therefore, data from the 7th–9th waves were included.

#### Inclusion and Exclusion Criteria

**Figure 2:**
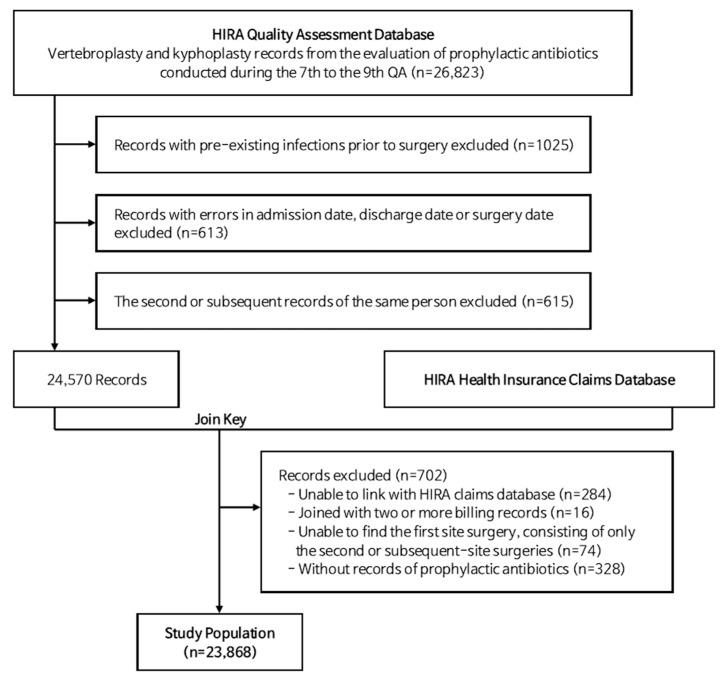
Flowchart of study population selection: 23,868 patients who underwent vertebroplasty or kyphoplasty were included after applying inclusion and exclusion criteria.

According to the QA program selection criteria, patients with documented preoperative infections were excluded from the analysis. Specifically, exclusion applied to patients who (1) received antibiotics for a confirmed infection or (2) had physician or infectious disease specialist records indicating a condition requiring antibiotic treatment prior to surgery. In addition, cases involving multiple operations during a single admission or concurrent spinal and other surgical procedures were excluded. After applying these criteria and resolving discrepancies between the QA and health insurance claims data, the final study population consisted of 23,868 patients from the 7th to 9th QA waves (Figure 2).

### 4.3. QA Criteria

#### 4.3.1. Types and Duration of Use of Antibiotics

The QA criteria for prophylactic antibiotic use in spinal surgery evolved substantially across the study period. In the 7th and 8th QA waves, the guidelines were relatively flexible: they discouraged, but did not strictly prohibit, the use of aminoglycosides, third-generation cephalosporins, or combination regimens. In contrast, the 9th QA wave adopted stricter standards, permitting only first- or second-generation cephalosporins, except in cases of documented allergies.

Furthermore, whereas the 7th and 8th waves did not impose a strict limit on the duration of antibiotic use, the 9th wave mandated discontinuation within 24 h postoperatively. Despite these changes in allowable agents and treatment duration, the requirement for the initial antibiotic dose remained consistent throughout all waves—administration within one hour prior to skin incision.

#### 4.3.2. Postoperative Surgical Site Infection

Postoperative infections, including both SSIs and non-SSIs, were systematically assessed and documented during the index hospitalization or within the three-month QA evaluation period following VP or KP. The diagnostic criteria for SSIs were based on standardized clinical, microbiological, and radiological findings [36], including:

(1) Purulent discharge from the incision site or drainage catheter.
(2) Positive bacterial cultures from the incision site, deep tissues, or internal organs obtained under aseptic conditions.
(3) Clinical signs such as fever >38 °C, localized pain, tenderness, erythema, or spontaneous wound dehiscence.
(4) Abscess or other infectious findings identified in deep tissue or organs through imaging or pathology.
(5) A clinical diagnosis of SSI made by a surgeon or infectious disease specialist.

### 4.4. Comorbidities and Medical History

Evaluating comorbidities and medical history is essential for identifying factors that predispose patients to postoperative infections [7,37]. However, the QA process alone does not allow for the comprehensive documentation of preexisting conditions and other clinical factors that may influence infection risk. To address this limitation, we supplemented the QA dataset with information from the national health insurance claims database, which was linked through anonymous join keys. This integration enabled extraction of relevant patient histories and provided a broader context for risk assessment. Our analysis incorporated covariates that could be reliably identified from the dataset and that overlapped with risk factors outlined in the Asia Pacific Society of Infection Control (APSIC) guidelines for the prevention of SSIs. In this way, we captured SSI-related risk factors within the inherent constraints of the available data [38].

To identify comorbidities, ICD-10 diagnostic codes were applied to patient records from the period prior to and during hospitalization for spinal surgery. The study included major comorbidities such as DM (E10–E14) and hypertension (I10–I15). Additionally, we evaluated conditions potentially associated with increased postoperative infection risk, including malnutrition (E40–E46), uncontrolled diabetes (E10.64, E11.64, E12.64, E13.64, E14.65), and skin or soft tissue infections (L00–L08).

### 4.5. Incidence of SSIs and Evaluation of Prophylactic Antibiotic Use

The primary objectives of this study were to determine the incidence of SSIs following VP and KP and to evaluate the appropriateness of prophylactic antibiotic administration in these procedures. Patients were categorized by the timing of antibiotic administration (preoperative-only, postoperative-only, and pre- and postoperative) to assess the effectiveness of each strategy. In addition, the appropriateness of prophylaxis was evaluated according to the type of antibiotic used. Particular attention was given to first- and second-generation cephalosporins, which are recommended by both international and national QA guidelines [27,28]. Less commonly prescribed agents were grouped as “others” because of their small sample sizes and clinical heterogeneity.

### 4.6. Statistical Analyses

Continuous variables were summarized as means with standard deviations and compared using Student’s *t*-test or the Mann–Whitney U test, as appropriate. Categorical variables were expressed as frequencies and percentages and compared using Pearson’s chi-square test or Fisher’s exact test. For comparisons involving more than two categories, post hoc analyses with Bonferroni correction were performed. A *p*-value < 0.05 was considered statistically significant.

Because SSIs were rare (overall incidence 0.20%) and no events occurred in the preoperative-only group, conventional logistic regression was affected by quasi-complete separation, producing unstable or infinite estimates. To address this issue, we applied Firth’s penalized likelihood logistic regression, which reduces small-sample bias and yields more reliable estimates under sparse data conditions. Results are presented as aORs with 95% CIs, with statistical significance inferred when the CI did not include 1.00 [39]. All analyses were performed using SAS software (version 7.1; SAS Institute, Cary, NC, USA) with the FIRTH option in PROC LOGISTIC.

## 5. Conclusions

This nationwide study provides important insights into prophylactic antibiotic use in VP and KP. Among 23,868 patients, the overall incidence of SSI was low (0.2%). Our findings suggest that a single preoperative dose of antibiotics, particularly first- or second-generation cephalosporins, may be sufficient to prevent postoperative infections, even in patients with higher baseline risk. Multivariate analysis further demonstrated that broader-spectrum or perioperative combination regimens offered no additional benefit in infection prevention. By contrast, undergoing surgery at a tertiary hospital and the presence of malnutrition were independently associated with increased SSI risk, emphasizing the importance of tailored infection control strategies for these vulnerable populations.

## Figures and Tables

**Table 1:** Incidence of postoperative infections in vertebroplasty and kyphoplasty patients (*N* = 23,868).

Variable	Total (*N* = 23,868)	Vertebroplasty (*N* = 20,835)	Kyphoplasty (*N* = 3033)	*p*-Value *
Surgical site infections	47 (0.20%)	43 (0.21%)	4 (0.13%)	0.387
Non-surgical site infections	364 (1.53%)	315 (1.51%)	49 (1.62%)	0.663
Total postoperative infections	407 (1.71%)	354 (1.7%)	53 (1.75%)	0.848

* Indicates the statistical difference in infection rates between vertebroplasty and kyphoplasty.

**Table 2:** Baseline characteristics of patients by timing of prophylactic antibiotic administration.

Variable	Preoperative-Only (*N* = 1131)	Postoperative-Only (*N* = 2529)	Both Preoperative and Postoperative (*N* = 20,208)	*p*-Value
Age (years), means (SD)	77.62 (8.48)	76.68 (8.37)	77.43 (8.38)	<0.001
Comorbidities				
Diabetes mellitus (DM)	388 (34.31%)	763 (30.17%)	6127 (30.32%)	0.017
Hypertension	589 (52.08%)	1060 (41.91%)	9738 (48.19%)	<0.001
History				
Malnutrition	44 (3.89%)	71 (2.81%)	606 (3.00%)	0.188
Uncontrolled DM	9 (0.80%)	13 (0.51%)	108 (0.53%)	0.497
Skin or soft tissue infection	52 (4.60%)	111 (4.39%)	1012 (5.01%)	0.349
Hospital type				
Tertiary	166 (14.68%)	9 (0.36%)	702 (3.47%)	<0.001
General	438 (38.73%)	322 (12.73%)	7216 (35.71%)	
Hospital	527 (46.60%)	2198 (86.91%)	12,290 (60.82%)	
Antibiotics used				
1st or 2nd generation cephalosporin only	1072 (94.78%)	2209 (87.35%)	18,098 (89.56%)	<0.001
Other antibiotics only	38 (3.36%)	91 (3.60%)	672 (3.33%)	
1st or 2nd generation cephalosporin and other antibiotics	21 (1.86%)	229 (9.05%)	1438 (7.12%)	
Surgery type				
Vertebroplasty	930 (82.23%)	2223 (87.90%)	17,682 (87.50%)	<0.001
Kyphoplasty	201 (17.77%)	306 (12.10%)	2526 (12.50%)	
Operation time (minutes), median (Q1–Q3)	25.00 (15.00–34.00)	30.00 (20.00–40.00)	25.00 (15.00–25.00)	<0.001
Total hospitalization days, median (Q1–Q3)	3.00 (2.00–10.00)	5.00 (3.00–12.00)	6.00 (3.00–14.00)	<0.001

**Table 3:** Incidence of postoperative infections by timing of prophylactic antibiotic administration.

Variable	Preoperative-Only (*N* = 1131)	Postoperative-Only (*N* = 2529)	Both Preoperative and Postoperative (*N* = 20,208)	*p*-Value
Surgical site infections	0 (0.00%) ^a^	9 (0.36%) ^b^	38 (0.19%) ^c^	0.069
Non-surgical site infections	13 (1.15%) ^a,b^	20 (0.79%) ^a^	331 (1.64%) ^b^	0.003
Total postoperative infections	13 (1.15%) ^a,b^	29 (1.15%) ^a^	365 (1.81%) ^b^	0.018

Identical superscript characters indicate no significant differences between groups in the post hoc analysis.

**Table 4:** Incidence of postoperative infections by type of prophylactic antibiotics used.

	Ceph 1st/2nd (+), Others (−) (*N* = 21,379)	Ceph 1st/2nd (−), Others (+) (*N* = 801)	Ceph 1st/2nd (+), Others (+) (*N* = 1688)	*p*-Value
Surgical site infection	35 (0.16%) ^a^	3 (0.37%) ^a,c^	9 (0.53%) ^c^	0.004
Non-surgical site infection	122 (0.57%) ^a^	22 (2.75%) ^b^	220 (13.03%) ^c^	<0.001
Total postoperative infection	156 (0.73%) ^a^	25 (3.12%) ^b^	226 (13.39%) ^c^	<0.001

Identical superscript characters indicate no significant differences between groups in the post hoc analysis.

**Table 5:** Multivariate logistic regression analysis of risk factors for surgical site infections.

Predictors	Reference Category	Surgical Site Infections Odds Ratio (95% Confidence Intervals)
Group		
Preoperative-only	Both preoperative and postoperative	0.270 (0.021–3.554)
Postoperative-only	Both preoperative and postoperative	24.806 (1.208–509.193) *
Age		1.011 (0.976–1.048)
Sex		
Male	Female	1.679 (0.852–3.308)
QA period		
7th and 8th QA	9th QA	0.095 (0.041–0.218) *
Antibiotics used		
With usage of 1st or 2nd generation cephalosporin	Without usage of 1st or 2nd generation cephalosporin	0.921 (0.252–3.364)
Insurance types		
Medical aids	Health insurance coverage	1.709 (0.739–3.956)
Hospital types		
Tertiary	Hospital	3.566 (1.017–12.502) *
General	Hospital	1.149 (0.605–2.182)
Comorbidity/Medical history		
Diabetes mellitus, Yes	No	0.718 (0.374–1.379)
Hypertension, Yes	No	1.027 (0.558–1.888)
Malnutrition, Yes	No	2.915 (1.129–7.522) *
Allergy to antibiotics		
Presence	Absence	3.521 (0.650–19.058)
Surgery types		
Kyphoplasty	Vertebroplasty	0.597 (0.217–1.646)
The number of concomitant spine surgeries		0.897 (0.458–1.757)
Operation time (minutes) †		1.311 (0.755–2.276)
Total antibiotics administration time (days) †		2.147 (1.506–3.061) *

† Calculated as log-transformed values due to exponential distribution; max-rescaled R-Square = 0.138; variables with statistically significant associations (confidence interval not including 1.00) are marked with an asterisk.

## Data Availability

Original data will be made available upon reasonable request.

## Supplementary Materials

The following supporting information can be downloaded at: https://www.mdpi.com/article/10.3390/antibiotics14090901/s1. Table S1. Baseline characteristics of patients by timing of prophylactic antibiotic administration. Table S2. Distribution of Prophylactic Antibiotic Classes by Administration Timing.

## Abbreviations

The following abbreviations are used in this manuscript:

VP	Vertebroplasty
KP	Kyphoplasty
SSIs	Surgical site infections
HIRA	Health Insurance Review and Assessment
QA	Quality assessment
DM	Diabetes mellitus
OR	Odds ratio
CI	Confidence interval
NASS	North American Spine Society
ICD-10	International Classification of Diseases, 10th Revision
