# Supplementary material for: Prophylactic Antibiotics in Vertebroplasty and Kyphoplasty: A Nationwide Analysis of Infection Rates and Antibiotic Use in South Korea
Source: Antibiotics (Basel). 2025 Sep 5;14(9):901. doi: 10.3390/antibiotics14090901 (PMC12466463; doi:10.3390/antibiotics14090901)
Supplement: Supplementary file 1 [file antibiotics-14-00901-s001.zip › antibiotics-3798522-supplementary.pdf]

Supplement Table S1. Baseline characteristics of patients by timing of prophylactic antibiotic administration.

Variable	Preoperative-only (N=1,131)	Postoperative-only (N=2,529)	Both preoperative and postoperative (N=20,208)	*P*-value
Age (years), means (SD)	77.62 (8.48)	76.68 (8.37)	77.43 (8.38)	<0.001
Wave				
7th	332 (29.35%)	740 (29.26%)	5,412 (26.78%)	<0.001
8th	132 (11.67%)	946 (37.41%)	7,112 (35.19%)	
9th	667 (58.97%)	843 (33.33%)	7,684 (38.02%)	
Sex				
Male	205 (18.13%)	423 (16.73%)	3,225 (15.96%)	0.110
Female	926 (81.87%)	2,106 (83.27%)	16,983 (84.04%)	
Comorbidities*				
Diabetes mellitus (DM)	388 (34.31%)	763 (30.17%)	6,127 (30.32%)	0.017
Hypertension	589 (52.08%)	1,060 (41.91%)	9,738 (48.19%)	<0.001
History*				
Malnutrition	44 (3.89%)	71 (2.81%)	606 (3.00%)	0.188
Uncontrolled DM	9 (0.80%)	13 (0.51%)	108 (0.53%)	0.497
Skin or soft tissue infection	52 (4.60%)	111 (4.39%)	1,012 (5.01%)	0.349
Insurance type				
Health insurance coverage	1,008 (89.12%)	2,315 (91.54%)	18,534 (91.72%)	0.009
Medical aids	123 (10.88%)	214 (8.46%)	1,674 (8.28%)	
Hospital type				
Tertiary	166 (14.68%)	9 (0.36%)	702 (3.47%)	<0.001
General	438 (38.73%)	322 (12.73%)	7,216 (35.71%)	
Hospital	527 (46.60%)	2,198 (86.91%)	12,290 (60.82%)	
Antibiotics used				
1st or 2nd generation cephalosporin only	1,072 (94.78%)	2,209 (87.35%)	18,098 (89.56%)	<0.001
Other antibiotics only	38 (3.36%)	91 (3.60%)	672 (3.33%)	
1st or 2nd generation cephalosporin and other antibiotics	21 (1.86%)	229 (9.05%)	1,438 (7.12%)	
Surgery type				
Vertebroplasty	930 (82.23%)	2,223 (87.90%)	17,682 (87.50%)	<0.001
Kyphoplasty	201 (17.77%)	306 (12.10%)	2,526 (12.50%)	
Allergy to antibiotics, presence	10 (0.88%)	43 (1.70%)	250 (1.24%)	0.072
Number of surgical codes billed per procedure, mean (SD)	1.23 (0.45)	1.12 (0.34)	1.16 (0.40)	<0.001
Operation time (minutes), median (Q1-Q3)	25.00 (15.00-34.00)	30.00 (20.00-40.00)	25.00 (15.00-25.00)	<0.001
Total hospitalization days, median (Q1-Q3)	3.00 (2.00-10.00)	5.00 (3.00-12.00)	6.00 (3.00-14.00)	<0.001

Supplement Table S2. Distribution of Prophylactic Antibiotic Classes by Administration Timing

Drug class	Included antibiotics	Preoperative only (N=1,131)	Postoperative only (N=2,529)	Both preoperative and postoperative (N=20,208)
**1st generation cephalosporin**	cefazolin, cefradine, cefadroxil, ceftezole, cephalexin, cephazedone, cefroxadine	802 (70.91%)	1,982 (78.37%)	15,119 (74.82%)
**2nd generation cephalosporin**	cefaclor, cefotiam, cefuroxime, cefminox, cefprozil, flomoxef, cefmetazole, cefotetan, cefbuperazone, cefoxitin, cefamandole	364 (32.18%)	1,369 (54.13%)	8,357 (41.35%)
**3rd generation cephalosporin**	ceftazidime, cefotaxime, ceftriaxone, cefixime, ceftizoxime, cefpodoxime, cefodizime, cefditoren, cefdinir, ceftibuten, cefcapene, cefpiramide, cefoperazone, cefetamet	20 (1.77%)	186 (7.35%)	905 (4.48%)
**4th generation cephalosporin**	cefepime	0 (0.00%)	0 (0.00%)	6 (0.03%)
**Aminoglycoside**	tobramycin, netilmicin, amikacin, isepamicin, arbekacin, gentamicin, ribostamycin	10 (0.88%)	50 (1.98%)	283 (1.40%)
**Penicillin**	amoxicillin, ampicillin, nafcillin, piperacillin, ticracillin, sultamicillin	12 (1.06%)	25 (0.99%)	396 (1.96%)
**Quinolone**	levofloxacin, ciprofloxacin, moxifloxacin, norfloxacin, tosufloxacin, balofloxacin, ofloxacin, gemifloxacin, lomefloxacin	19 (1.68%)	69 (2.73%)	713 (3.53%)
**Glycopeptides**	vancomycin, teicoplanin	0 (0.00%)	0 (0.00%)	14 (0.07%)
**Etc.**	rifampicin, isoniazid, pyrazinamide, ethambutol, metronidazole, ornidazole, meropenem, doripenem, imipenem, ertapenem, fusidate, fosfomycin, lincomycin, clindamycin, roxithromycin, clarithromycin, azithromycin, aztreonam, linezolid, colistin, sulfamethoxazole, doxycycline, minocycline, tigecycline	2 (0.18%)	8 (0.32%)	123 (0.61%)

The “Etc.” category includes carbapenems, macrolides, glycopeptides, and other less frequently used antibiotics.
